# The Dysbiosis of Gut Microbiota Caused by Low-Dose Cadmium Aggravate the Injury of Mice Liver through Increasing Intestinal Permeability

**DOI:** 10.3390/microorganisms8020211

**Published:** 2020-02-05

**Authors:** Yehao Liu, Yuhui Li, Yuhong Xia, Kaiyong Liu, Lingling Ren, Yanli Ji

**Affiliations:** 1 School of Public Health, Anhui Medical University, Hefei 230032, Anhui, China; liuyehao@ahmu.edu.cn (Y.L.); liukaiyong163@163.com (K.L.); linglren@ahmu.edu.cn (L.R.); 2 Department of Biological and Environmental Engineering, Hefei University, Hefei 230032, Anhui, China; yuhuili112@163.com

**Keywords:** transcriptome analyses, gut microbiota, cadmium, intestinal permeability

## Abstract

Cadmium (Cd), widely present in food and drinking water at low doses, can cause health risks. However, the mechanistic effects of long-term Cd exposure at low dose through dietary intake is poorly studied. The aim of this study is to elucidate whether the dysbiosis of gut microbiota caused by Cd at an environmental low dose can aggravate the injury of mice liver, and the possible mechanism is investigated. In order to explore the potential underlying mechanism, the analyses of the variation of gut microbiota composition, intestinal permeability, and hepatic transcriptome were conducted. Our results showed that gut microbiota was disturbed. The rise of intestinal permeability induced by the dysbiosis of gut microbiota resulted in more Cd ions accumulating in mice liver, but it could be restored partly through depleting gut microbiota by antibiotics cocktail. Transcriptomic analyses indicated that 162 genes were significantly differentially expressed including 59 up-regulated and 103 down-regulated in Cd treatment. These genes were involved in several important pathways. Our findings provide a better understanding about the health risks of cadmium in the environment.

## 1. Introduction

Cd, a non-essential toxic metal, is widely distributed in soils, air dust and water. It can produce multiple health risks even at environmental low dose [1,2]. Nowadays, the main entrance of Cd into human body is in diets, especially in grains and vegetables from contaminated soils and waters [3]. For instance, rice, a major staple food and the most widely consumed cereal grain in Asia, has been become a main source of Cd entry to the human body. It has been reported that 88% of rice grain from south China exceed the Chinese maximum permissible limit for Cd (0.1 mg fresh weight kg^−1^). The dietary Cd intake varies from 66.5 to 116 μg Cd kg^−1^ body weight month^−1^ in Hunan Province which are over 2.7 times the tolerable dietary intake of 25 μg Cd kg^−1^ body weight month^−1^ recommended by the Joint Food and Agriculture Organization of the United Nations and World Health Organization (FAO/WHO) Expert Committee on Food Additives [4,5]. After dietary absorption, Cd can remain in the human body with a half-life of 10–30 years. In the human body, liver is one of the main deposits for Cd [6]. Since liver runs many essential functions and plays a major role in the regulation of nutrient metabolism, the adverse effects of Cd exposure on the functional disorders of liver have been deeply investigated. Some chronic liver diseases, such as non-alcoholic fatty liver disease (NAFLD) [7,8], hepatic necroinflammation, are associated with the Cd burden. Go et al. report that 10 mg L^−1^ of Cd in the drinking water causes hepatic metabolic changes [9]. Apart from that, several studies based on transcriptional analyses discovered that the expression of some hepatic genes is altered under Cd toxicity in animals [9,10,11]. These genes are involved in transcriptional regulation, cell proliferation, stress response, apoptosis, nutrition metabolism. However, the health risk at low-level exposure and its underlying mechanism need further study.

It has been confirmed that gut microbiota is involving in a host’s health throughout life [12]. Its functions include the production of nutrients, immune response, and protection from infections [13,14]. However, the dysbiosis of gut microbiota increases intestinal permeability and drives inflammation and mortality [15,16]. Exposure to chemicals, even at the low doses recognized as safe, can disturb gut microbiota and result in metabolic disorders in the future [17]. Moreover, increased intestinal permeability allows more toxins into the body. Therefore, gut microbiota play a vital role in assessing the toxicity of environmental pollutants, particularly the trace heavy metals such as Cd. According to the theory of the gut–liver axis, gut heath is implicated in the development and progression of liver diseases [18,19], but little recent evidence exists that a low dose of Cd can modulate some intestinal functions, and eventually cause abnormal hepatic functions.

This study aimed at clarifying the linkage of the composition of gut microbiota, gut barrier functions and hepatic transcriptome after long-term exposure of low-dose Cd. We hypothesized that the dysfunction of the gut barrier caused by the altered gut microbiota imposed more damage on mice livers.

## 2. Materials and Methods

### 2.1. Animals

Animal experiments were approved (no. 20180293, approved date: 18/03/2018) and carried out according to Anhui Medical University’s Standing Committee On animals (Anhui, China). Female C57BL/6J mice (22 g, 6 weeks old) were purchased from the Beijing Vital River Laboratory Animal Technology Co. Ltd. (Beijing, China). Mice were housed in cages for a 12 h light/dark cycle at 24 °C with water and food ad libitum. After one-week acclimation, a total of 30 mice were randomly divided into three groups comprising 10 mice each. A control group received pure drinking water and a low Cd treatment group received drinking water containing 10 mg L^−1^ cadmium chloride (99.99% purity, Aladdin, Shanghai, China). In order to evaluate whether the presence of gut microbiota affected intestinal permeability, the third group received drinking water containing 10 mg L^−1^ cadmium chloride and antibiotic cocktail. It was defined as Cd + antibiotic treatment. For the depletion of gut microbiota, mice were treated by the antibiotic cocktail and one antifungal which was described by Zarrinpar et al. [20] with slight modifications. Briefly, the antibiotic cocktail comprised four antibiotics (ampicillin 10 mg kg^−1^, vancomycin 5 mg kg^−1^, metronidazole 10 mg kg^−1^, neomycin 10 mg kg^−1^) and amphotericin B (0.1 mg kg^−1^). In order to mitigate the harm caused by the antibiotic cocktail, mice drank the antibiotic cocktail for 7 consecutive days then switched to distilled water for the next 4 consecutive days. This procedure was repeated until the end of the experiment. The experiment was run for 52 weeks until the Cd level was stable in the mice organs.

### 2.2. Intestinal Permeability In Vivo

After a 12 h fast, mice were gavaged with 0.8 mg g^−1^ body weight of fluorescein-isothiocyanate-dextran (FITC-dextran, FD4, 3000–5000 kD, Sigma-Aldrich, St. Louis, MI, USA) in a volume of 0.2 mL. Blood samples were collected by cardiac puncture at 4 h after administration of FITC-dextran. These samples were centrifuged at 3000 rpm for 5 min at room temperature to obtain serum. The fluorescence intensity in serums was determined according to the standard curve which was constructed by the serial dilution of FITC-dextran.

### 2.3. RNA Extraction and Quantitative Polymerase Chain Reaction (qPCR) Amplification of the Genes for Tight-Junction Proteins

Ileum samples from the control group, low Cd treatment and antibiotic treatment were collected to extract total RNA using an Ultrapure RNA kit (CWbio, Beijing, China) according to the manufacturer’s protocol. Complementary DNA (cDNA) was synthesized by reverse transcription using the EasyQuick RT MasterMix kit (CWbio, Beijing, China). The expression of zonula occludens 1 (ZO-1), junctional adhesion molecule A (JAM-A), occluding was quantified by quantitative polymerase chain reaction (qPCR). Briefly, the primers used to amply these genes are list in Table S1, qPCR was performed on LightCycler 96 (Roche, Santa Clara, CA, USA) in a 50-μL reaction mixture containing 25 μL of 2× UltraSYBR Mixture (CWbio, Beijing, China), 0.3 μM of each primer, and cDNA as a template. The ratio of gene expression was recorded as the fold change in expression between the treated samples and the control group. The results were normalized to the glyceraldehyde-3-phosphate dehydrogenase (GAPDH) housekeeping gene. Each sample was run in triplicate.

### 2.4. Quantitative PCR Amplification of Akkermansia Muciniphila and the Overall Microbe Census

The abundance of microbes and *A. mucinipbila* was determined by qPCR. Briefly, the day before euthanasia, about 0.2 g fresh feces were collected from each mouse. Total DNA was extracted using QIAamp Fast DNA Stool Mini Kit (QIAGEN GmbH, Hilden, Germany) according to the manufacturer’s protocol and quantified using a Nanodrop 2000 spectrophotometer (Thermo Scientific, Waltham, MA, USA). The primers used to amply *A. mucinipbila* and the overall microbe census are listed in Table S1. The detailed information of qPCR amplification was described in our previous experiment [21].

### 2.5. High-Throughput RNA Sequencing

Total RNA was extracted from fresh mice livers (*n* = 3 for each group) with an Ultrapure RNA Kit (CWbio, Beijing, China) according to the manufacturer’s protocol. The purity and concentration of RNA was assessed by Nanodrop 2000 spectrophotometer (Thermo Scientific, Waltham, MA, USA). The library construction and RNA-seq were conducted by Shanghai Majorbio Com. Ltd. (Shanghai, China). Briefly, the RNA-seq libraries were constructed using the Illumina TruSeq RNA Sample Preparation Kit (Illumina, San Diego, CA, USA) according to the manufacturer’s protocol. Sequencing was carried out using an Illumina Hiseq Xten platform. The level of gene expression was determined using reads per kilobase per million (RPKM) and analyzed using the HTSeq software. A cut-off value of RPKM mapped reads greater than 1 was used to define the gene expression. The analysis of differentially expressed genes (DEGs) was performed using DESeq software (version 1.36.0, Boston, MA, USA), and the fold change and Fisher-test were used to choose DEGs. The false discovery rate (FDR) was used to adjust *p*-value. The DEGs with *p*-value lower than 0.05 and FDR lower than 0.02 were considered to be statistically significant. The enrichment analysis of DEGs was performed using the Kyoto Encyclopedia of Genes and Genomes (KEGG) database which is dealing with genomes, biological pathways, and disease. The online Path-Finder software (http://www.genome.jp) was used in KEGG enrichment analysis.

Quantitative PCR was also performed to confirm the DEGs obtained from the RNA-Seq results. Five DEGs were randomly selected and examined using qPCR with protocol being described above. The primer sequences were listed in Table S1. The 2^−∆∆*C*t^ method was used for the quantification with the GAPDH housekeeping gene as reference gene.

### 2.6. The Analyses of Gut Microbiota

Total bacterial genomic DNA was extracted from fresh fecal pellets using a QIAamp Fast DNA Stool Mini Kit (QIAGEN GmbH, Hilden, Germany) according to the manufacturer’s protocol and quantified using a Nanodrop 2000 spectrophotometer (Thermo Scientific, Waltham, MA, USA). The bacterial 16S rRNA gene was amplified with the 341F and 806R primers (the sequence of primers is list in Table S1) targeting the V3–V4 region. All PCR amplifications were conducted in triplicate for each treatment. The PCR products were purified using the Gel Extraction Kit (CWbio, Beijing, China) according to the manufacturer’s instructions and quantified using Nanodrop 2000 (Thermo Scientific, Waltham, MA, USA). Purified amplicons were pooled together in equal amounts and paired-end sequenced on the platform of Illunina Miseq (Illumina, San Diego, CA, USA). Raw sequence reads were quality-filtered and processed using the Quantitative Insights Into Microbial Ecology (QIIME) program as described by Caporaso et al. [22,23]. Operational taxonomic units (OTUs) were delineated at 97% sequence similarity. Alpha and beta diversity indices were determined using QIIME. Linear discriminant analysis effect size (LEfSe) analysis was conducted with the online tool (https://huttenhower.sph.harvard.edu/galaxy).

### 2.7. Determination of Cd Concentration in Mouse Liver

The body weight of mice was determined prior to sacrifice by decapitation. Freshly liver samples were collected and weighted, two-fold volume of 65% HNO_3_ was added and the samples were digested with a high-performance microwave system. A solution prepared from the digested sample was used for determining the Cd level by graphite furnace atomic absorption spectrometry. The detailed information was described in our previous study [21].

### 2.8. Enzyme-Linked Immunosorbent Assay (ELISA)

To evaluate liver injury, the activities of alanine aminotransferase (ALT) and aspartate aminotransferase (AST) in freshly collected plasma were measured using a corresponding enzyme-linked immunosorbent assay (ELISA) kit (NJJCBio, Nanjing, China) following the manufacturer’s instructions.

### 2.9. Statistical Analysis

Results were presented as means ± standard deviation (SD). The significant differences were examined using analysis of variance (ANOVA) or Student’s *t*-test. Differences in the abundance of gut microbiota were analyzed by the Mann–Whitney *U* test, and differences with *p*-value < 0.05 were considered to be statistically significant

## 3. Results

### 3.1. Oral Low-Level Cd Causes the Alteration of Gut Microbiota

**Figure 1:**
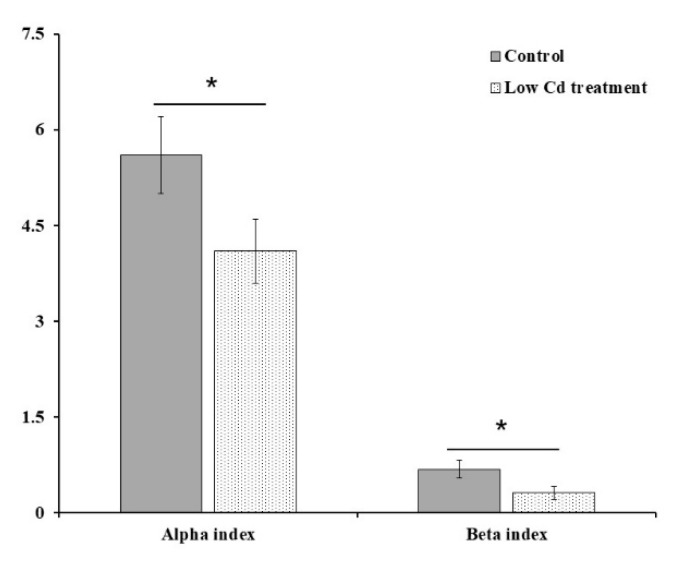
Alpha diversity and intragroup beta diversity of the control and low Cd treatment groups; * *p* < 0.05.

**Figure 2:**
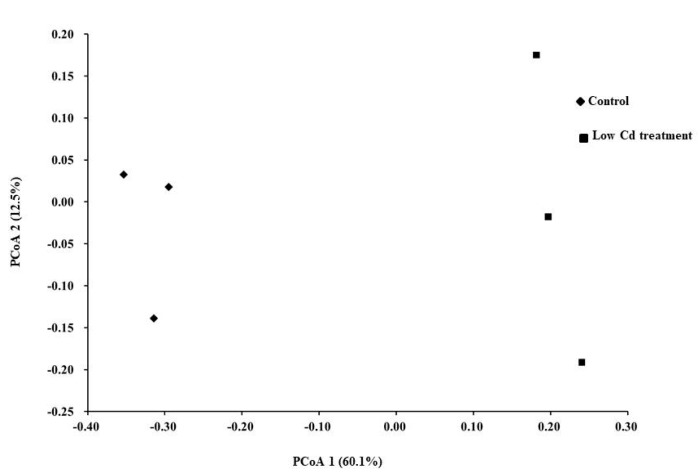
The plot generated by the weighted UniFrac-based PCoA.

**Figure 3:**
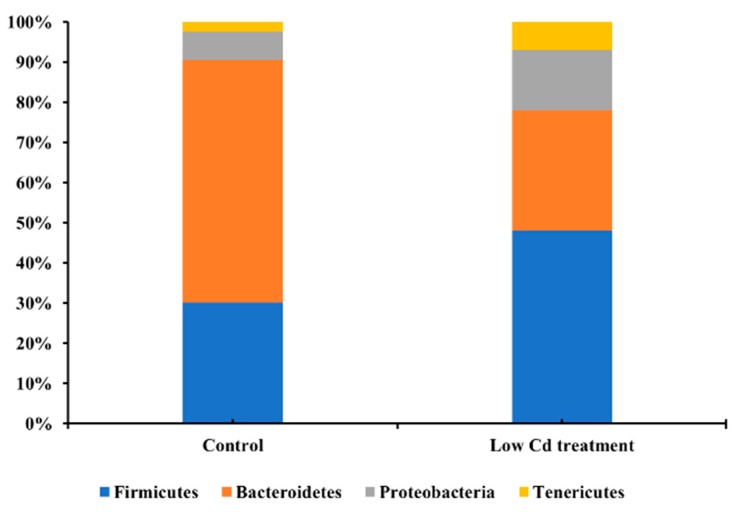
The relative abundance of predominant bacteria at the phylum level in the control and low Cd treatment groups.

Since Cd usually enter the host by food or drinking water, the composition of gut microbiota might be altered after contact with Cd in the digestive tract. Firstly, the effects of Cd exposure on gut microbiota were assessed based on 16S rRNA gene high-throughput sequencing data. The alpha diversity of microbial community represented by the Shannon index was decreased significantly by 26.8% in the low Cd treatment group. Gut microbiota in the low Cd treatment group also showed lower intragroup beta diversity than the control group, which was compared by weighted UniFrac distance (Figure 1). Principal coordinate analysis (PCoA) based on weighted UniFrac revealed distinct clusters of gut microbiota composition between the two groups, the profile of the low Cd treatment group was quite distinct from that of the control group (Figure 2). Notably, the analysis of taxonomic profiling revealed that the pattern of gut microbiota was altered by Cd which was shown in Figure 3. There was notable difference in microbiota abundance through the percent of reads assigned at phylum level. In particular, the low Cd treatment group was largely dominated by Firmicutes (48%), Bacteroidetes (30%) and Proteobacteria (15%); whereas the control group was dominated by Firmicutes (30%) and Bacteroidetes (60%). These results suggested that even low-level Cd treatment can induce the alteration of gut microbiota.

**Figure 4:**
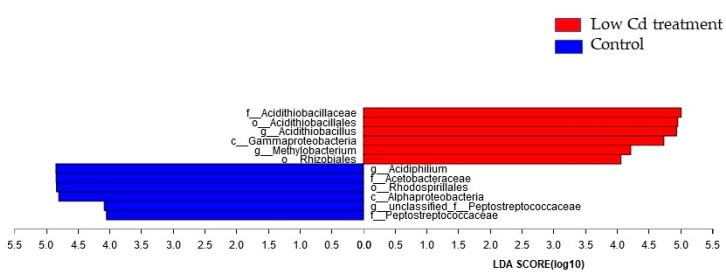
The most differentially abundant taxa between the control and low Cd treatment groups which was identified through the LDA score generated from linear discriminant analysis effect size (LEfSe) analysis.

Next, LEfSe comparison of the gut microbiota between the control group and the low Cd treatment group was conducted to determine the specific bacterial taxa associated with Cd exposure. As represented in the cladogram (Figure S1), the structure and predominant bacteria showed difference at different taxonomic levels. The significant difference in bacterial abundance from phylum to genus was compared via linear discriminant analysis (LDA) score. Most of the specific taxa were belonged to Proteobacteria and Firmicutes. Significant enrichment of taxa included Acidithiobacillaceae, Gammaproteobacteria, Methylobacterium and Rhizobiales (Figure 4).

### 3.2. Intestinal Permeability Is Increased after Cd Exposure

**Figure 5:**
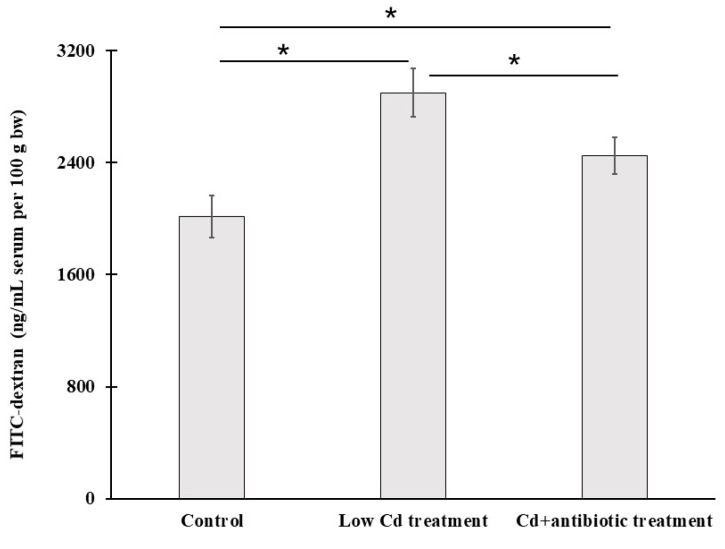
Fluorescein-isothiocyanate-dextran (FITC-dextran) levels in serum from the control and low Cd treatment groups as a measure of intestinal permeability. * *p* < 0.05.

Intestinal integrity plays a vital role in controlling the substance exchange. The biomarker of FITC-dextran level in serum is widely used to determine intestinal permeability. We evaluated intestinal permeability using this method too. Compared to the control group, the level of FITC-dextran in serum was increased significantly by 43.9% in the low Cd treatment group (Figure 5). Although the level of FITC-dextran in Cd + antibiotic treatment was still higher than the control group, it was significantly lower than the low Cd treatment group. These data indicated that the dysbiosis of gut microbiota induced by Cd could increase the level of FITC-dextran in serum, but the depletion of gut microbiota might contribute to the decrease of FITC-dextran concentration in serum. However, more evidence is needed to support this conclusion.

### 3.3. The Expression of Tight-Junction Proteins Is Closely Related to Intestinal Permeability

**Figure 6:**
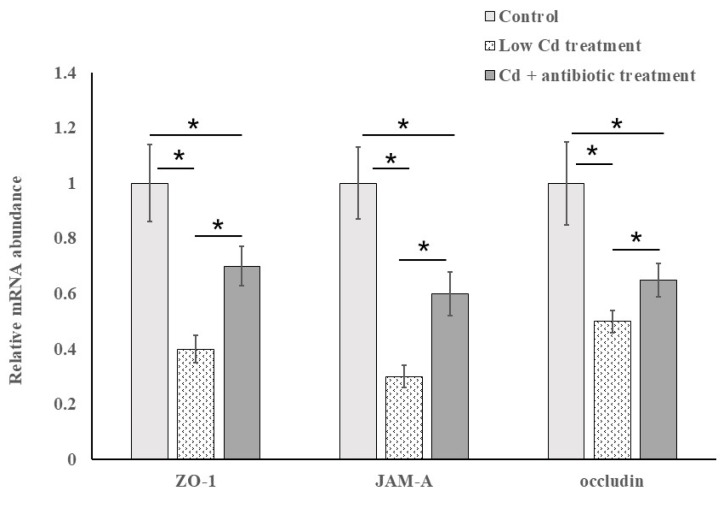
Levels of mRNA expression of the tight junction proteins from the control and low Cd treatment groups. Results are expressed as mean values (fold change respect to control). * *p* < 0.05.

ZO-1, JAM-A and occluding are intestinal tight-junction proteins. They are essential to maintain barrier function, and the stability of the tight junction. The low expression of tight-junction proteins has been proved to cause the increase of intestinal permeability. Thus, the expression of the three genes encoded these tight junction proteins was investigated to evaluate epithelial tight junctions. As shown in Figure 6, the expression of these three genes was significantly decreased in the low Cd treatment group when compared to the control group. After depletion of the gut microbiota by antibiotic cocktail, the expression of these three genes was restored partly although it was still lower than the control group.

### 3.4. The Abundance of *A. Mucinipbila* Is Negatively Correlated to Intestinal Permeability

**Figure 7:**
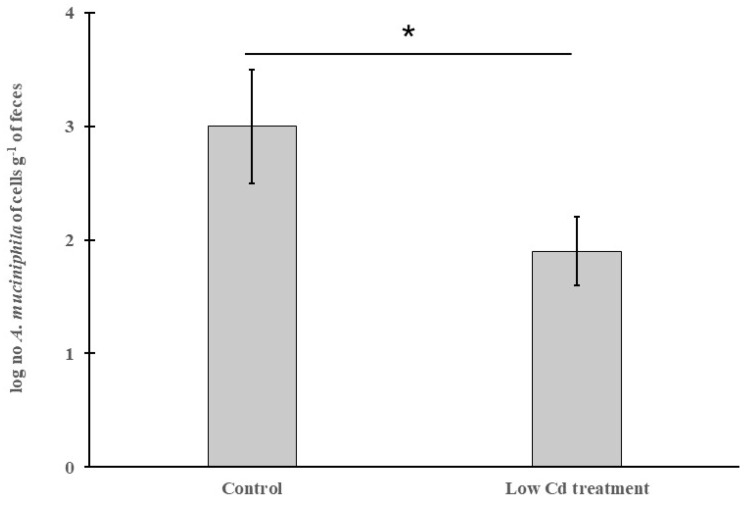
The relative abundance of *A. mucinipbila* in the control and low Cd treatment groups. * *p* < 0.05.

*A. mucinipbila* is a mucin-degrader in the human and animal gut microbiota, and its abundance has been proved to be linked with gut health, especially in keeping intestinal integrity. The result of applying qPCR to detect the abundance of *A. mucinipbila* is shown in Figure 7. The highest abundance of *A. mucinipbila* was in the control group. However, it was significantly decreased with about 36.7% in the low Cd treatment group. The toxicity of Cd may contribute to this phenomenon.

### 3.5. The Presence of Gut Microbiota Can Modulate Cd Level in the Liver

**Figure 8:**
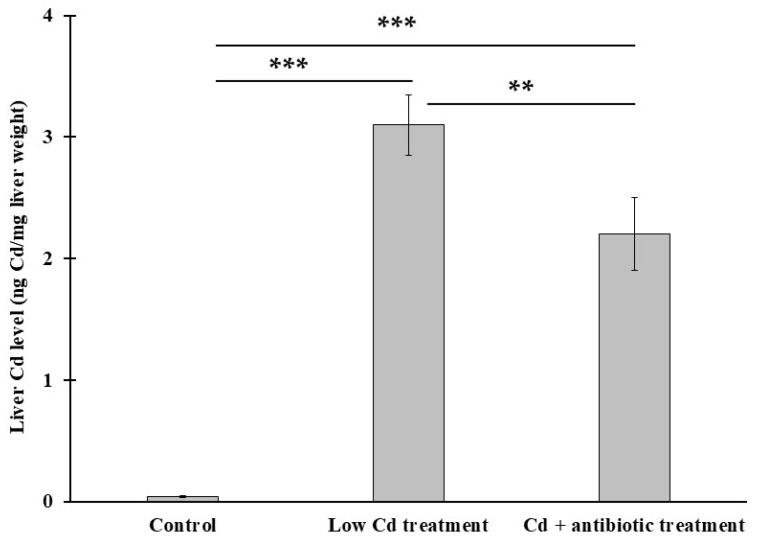
Cd concentration in mice livers. ** *p* < 0.01, *** *p* < 0.001.

One of the Cd toxicities on biological system is that it cannot be excreted effectively. In order to determine Cd level in liver, graphite furnace atomic absorption spectrometry was used in this study. Our results showed that Cd level was substantially higher in the low Cd treatment group (3.1 ± 0.16 ng Cd mg^−1^ liver) than the control group (Figure 8); however, it was decreased significantly to 2.4 ± 0.14 ng Cd mg^−1^ liver in Cd + antibiotic treatment which was significantly lower than that in the low Cd treatment group.

### 3.6. Mouse Liver Is Injured by Low-Level Cd

**Figure 9:**
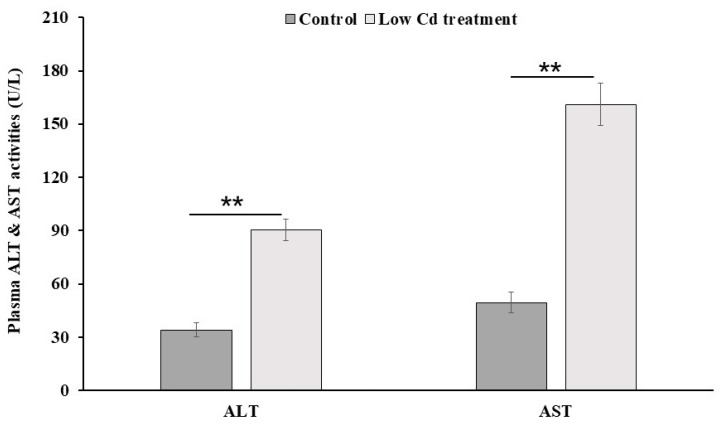
The activities of alanine aminotransferase (ALT) and aspartate aminotransferase (AST) in serum samples from the control and low Cd treatment groups. ** *p* < 0.01.

The deposition of Cd in mice livers can cause multiple damages. The level of liver enzymes measured in the peripheral blood provides a good estimate for liver injury. As shown in Figure 9, the level of ALT and AST was significantly higher in the low Cd treatment group than those in the control group (by 2.7 fold for ALT and 3.3 fold for AST). This finding suggested that even an environmental low dose of Cd can cause liver injury.

### 3.7. Cd Accumulation Induces Hepatic Genes Expression Differently

**Figure 10:**
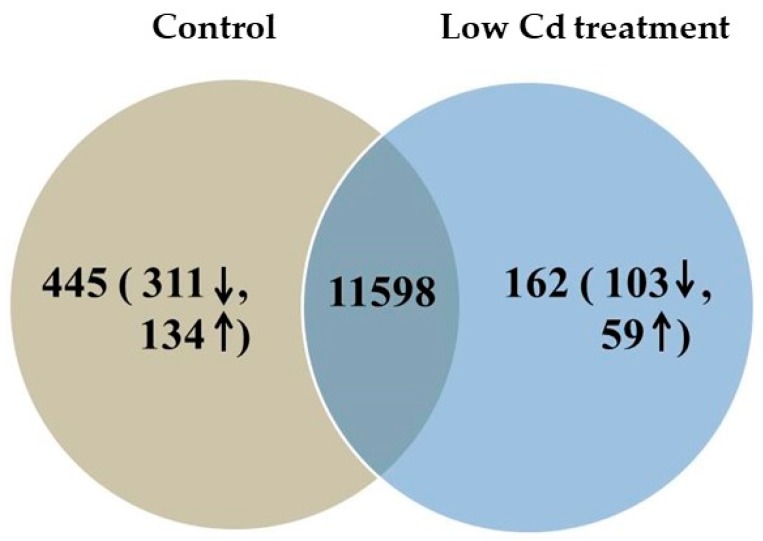
Venn diagram represents the numbers of overlapping genes between control and Low Cd treatment.

To further investigate the low-dose Cd induced injury to liver, we conducted the analyses of hepatic gene expression profile. After removing the unqualified reads, a total of 581.04 ± 33.84 and 563.01 ± 35.14 million clean reads were obtained for the control group and the low Cd treatment group, respectively. To evaluate the quality of the RNA sequencing data, the clean reads were mapped to the reference genome. Over 92% of the clean reads were mapped to the mouse reference genome. The detailed description for RNA-sequencing data is included in Table 1. The results showed that our RNA-sequencing data was reliable. Next, gene annotation and functional analysis were performed. The identified genes were aligned with several public databases, such as Gene Ontology (GO), KEGG and EggNOG (Evolutionary Genealogy of Genes: Non-supervised Orthologous Groups). Most of the genes were annotated using the GO database, followed by KEGG database. Subsequently, the DEGs were identified and defined as the genes with RPKM ratio greater than two folds. As shown in Figure 10, a total of 12,043 and 11,760 DEGs were identified in the control and low Cd treatment groups, respectively. In these DEGs, there were 445 and 162 DEGs uniquely expressed in the control and low Cd treatment groups, respectively, 11,598 DEGs were commonly expressed in both of the two treatments. Among the 162 unique DEGs in the low Cd treatment group, 59 genes were upregulated and 103 genes were downregulated. To test the reliability of RNA-sequencing results, five DEGs were randomly selected and examined using qPCR (Figure S2). The results showed that the mRNA level of *Acsf2*, *Gm15922*, *Itgal* and *Mvd* was decreased and *Fam35a* was increased in the low Cd treatment group, which were consistent with the RNA- sequencing results. It indicated that the RNA sequencing data in this study was reliable.

**Figure 11:**
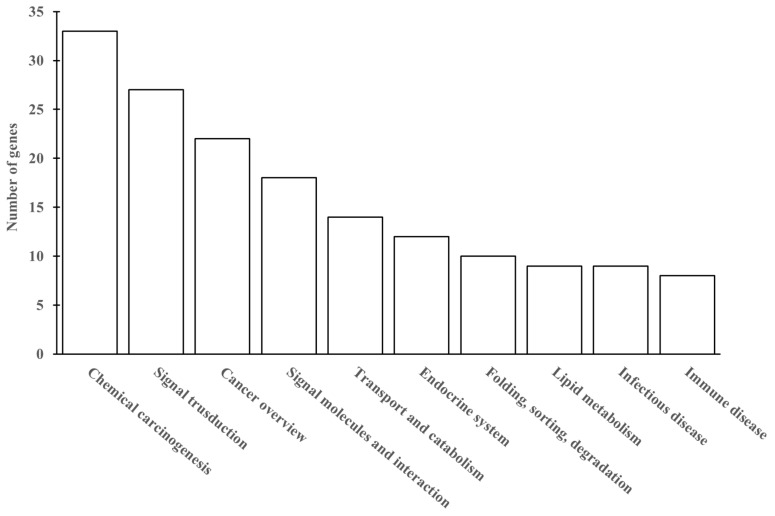
Kyoto Encyclopedia of Genes and Genomes (KEGG) database enrichment classification of differentially expressed genes (DEGs).

Finally, KEGG enrichment analysis of DEGs was performed to further investigate the hepatic injury induced by low-dose Cd. As shown in Figure S3, the DEGs between the control group and the low Cd treatment group were categorized into three types. The first type was related to the growth and modulation of hepatocytes, the second type dealt with the functions of cells and organelles, the third type was associated with binding activity and catalytic activity. The GO analysis of the genes regulated by low-dose Cd indicated that most of DEGs were involved in metabolic processes and provided protection against chemicals in the liver. Meanwhile, 162 DEGs were involved in the top 10 KEGG pathways (Figure 11). The most abundant groups were chemical carcinogenesis, signal transduction and cancer overview.

## 4. Discussion

In this study, a mouse model was established to analyze whether the composition of gut microbiota could be altered by environmental low-dose Cd exposure and its effect on intestinal permeability. Subsequently, cumulant Cd and its effects on the change of metabolic pathways in the liver at the gene level were investigated. The results demonstrated that the composition of gut microbiota was altered. It resulted in the increase of intestinal permeability and allowed more Cd to accumulate in the liver. Finally, the normal functions of multiple metabolic pathways were affected according to the results of transcriptome analyses.

Previous studies have demonstrated that Cd is toxic to the kidney and bones, and low-dose Cd exposure is also associated with some adverse effects such as cancers, diabetes, hypertension and some other chronic diseases [1,8,10]. However, the Cd dosages used in most studies are higher than the human intake through drinking water or diets [24,25]. It is not suitable to evaluate the Cd toxicity under the environmental low dose. We used 10 mg L^−1^ cadmium chloride in drinking water which is close to the mean intake by humans. Cd accumulation in the liver is very slow especially for low doses. Its effects on the liver is hard to be evaluated exactly in a short term because liver only receive about 15% amount of total intake of Cd [6]. The period of experiment was extended to one year in this study. The level of Cd in mouse liver was almost steady. As a result, our experimental design was reasonable. Our results indicated that such low dose of Cd can still alter gut microbiota and hepatic gene expression. It implies a possible universal impact for heavy metals.

Our findings showed that low Cd treatment reduced the diversity of the gut microbiota. However, some microbes including Acidithiobacillaceae, Gamma-proteobacteria, Methylobacterium and Rhizobiales became prevalent. This phenomenon has been found in many studies. For instance, most of Gamma-proteobacteria are found to be resistant to heavy metals [26,27]; some Rhizobiales belong to halophilic bacteria [28]; Acidithiobacillaceae and Methylobacterium are well known for being able to live in environments containing a high level of metal ions [29,30,31]. So, it is reasonable to detect these microbes in low Cd treatment for their resistance to environmental pressure. Since these microbes with high resistance to metal ions could grow well under low Cd treatment, the balance of gut microbiota was disrupted. It has been widely accepted that the disruption of gut microbiota can cause metabolic diseases, including obesity, insulin resistance, etc. [32,33]. On the other hand, Cd intake through drinking water or diet has two destinies; one is staying in the host’s intestinal tract which will exert an influence on gut microbiota, then is excreted with feces; the other is that Cd is absorbed by the host which will deposit in some organs including kidney, liver lung etc. However, there are few studies that have investigated the effects of gut microbiota disruption on the rate of Cd absorption by host’s organs. Our study uncovers that the disrupted gut microbiota caused by low-dose Cd affect the rate of host absorbing Cd. These results will provide insights to evaluate how gut microbiota puts its effects on host health.

To evaluate the effects of low-dose Cd exposure on intestinal integrity, the concentration of FITC-dextran in serum is measured. This method is widely used to determine intestinal integrity for its convenience and non-injury to mice [34]. A significant increase of FITC-dextran level in the serum from the low Cd treatment group was detected. It suggested that intestinal integrity was broken. The intestinal epithelium forms a selectively permeable barrier which is vital in preventing pathogenic invasion. Tight junctions are important in keeping intestinal barrier normal and are responsible for its integrity. Occludin, clandin-1 and ZO-1 are tight-junction proteins with different molecular structures and functions. Occludin and clandin-1 are transmembrane proteins which regulate the tight junction’s function and integrity; ZO proteins have been proved to play an important role in regulating the assembly of tight junctions. It has been confirmed that the variation in the gene expression of tight-junction proteins is associated with intestinal permeability [35,36]. In our study, the effects of the altered gut microbiota on gene expression of these three tight-junction proteins were evaluated. The results showed that the expression of the genes encoding occludin, clandin-1 and ZO-1 was significantly decreased. However, the depletion of gut microbiota by administration of an antibiotic cocktail caused an increase in gene expression of these tight-junction proteins. Meanwhile, some studies based on animal models have shown that extracellular vesicles derived from *A. muciniphila* have protection for the maintenance of intestinal homeostasis [37]. This microbe is essential in keeping mucus layer health in terms of mucus production and thickness [38]. We observed a reduction for this species in the low Cd treatment group. This result is in line with previous studies and is consistent with our qPCR data for tight-junction proteins [39]. These results indicate that the gut microbiota can modulate the transcription of these proteins and, in turn, regulate intestinal permeability.

Gene expression data indicated that 59 genes were increased and 103 genes were decreased in the low Cd treatment group when compared with the control group. According to the functions of identified Cd-regulated genes, the DEGs were associated with the liver injury including chemical carcinogenesis and signal transduction which are summarized in Figure 11. These results provided evidence that mouse liver was injured by the exposure of environmental low-dose Cd.

According to the results of transcriptome analyses, we found that the exposure of low-dose Cd caused liver injury by affecting the normal functions of several important pathways which could induce abnormal expression of various proteins. The GO analysis of the DEGs from the low Cd treatment group indicated that many of DEGs participated in the physiological process of stress resistance in the liver, mainly associated with biological processes and cellular components. In biological process, DEGs were mainly focused on cellular process, biological regulation and respond to stimulus. In cellular components, DEGs were mainly focused on organelle and cell membranes. In addition, these DEGs also contained some genes encoding catalytic activity of enzymes and binding. Thus, low-dose Cd could activate some enzymes to enhance Cd binding to liver cells. The result of GO enrichment analysis indicated that low-dose Cd could regulate various metabolic processes, such as inducing the defense response of liver cells, enhancing response to stress and the chemokine-mediated signaling pathway, and G-protein coupled chemoattractant receptor activity. Some other studies also have demonstrated that these metabolic processes are involved in Cd resistance. For instance, Huang et al. found one of chemokines, TGF-β1, could ameliorate Cd-induced nephrotoxicity [40]. Meanwhile, Huff et al. showed that arsenate and Cd could activate G-protein coupled estrogen receptor signaling in human lung adenocarcinoma cells [41]. Our results are consistent with these findings. However, these findings are obtained from a high dose of Cd treatment for a short time in mice models or a low dose of Cd treatment in cell lines. Our findings can extend the knowledge to Cd toxicity at an environmental low dose.

The activity of serum ALT and AST is widely used to evaluate liver function in animals and human beings [42,43]. We found a significant increase of the activity of these two enzymes in the low Cd treatment group. Cd deposition in liver may contribute to this phenomenon because Cd has been incriminated to induce hepatic dysfunction in many studies [8,9,10]. However, the underlying mechanisms still need to be investigated further. Combined with the results from transcriptome analyses, our findings suggested that multiple pathways in liver were altered by Cd, and it led to liver injury.

## 5. Conclusions

In summary, our findings showed that an oral low-dose Cd could cause disruption of gut microbiota, then an increase in intestinal permeability was obtained, and finally a disorder in hepatic gene expression occurred. The disruption of gut microbiota was a novel mechanism by which low-dose Cd could lead to negative consequences in the mouse liver. Moreover, decreased intestinal permeability could serve as a potential intervention to protect against the toxicity of heavy metals at an environmental low dose. In the future, we aim to verify the expression of proteins in all pathways affected by low-dose Cd in the liver and other organs, and deeply investigate the toxicity of low-dose Cd to gut microbiota.

## Figures and Tables

**Table 1:** Summary of RNA-sequencing data.

	Control	Low Cd Treatment
Total reads (×10^5^)	581.04 ± 33.84	563.01 ± 35.14
Total mapped reads	564.28 ± 35.34	545.29 ± 33.32
Mapped to reference genome %	92.76	93.26
Mapped to gene %	97.54	97.35
Mapped to exon %	95.45	95.61
Mapped to intergene %	2.35	2.71

## Supplementary Materials

The following are available online at https://www.mdpi.com/2076-2607/8/2/211/s1.
