# Supplementary material for: The Dysbiosis of Gut Microbiota Caused by Low-Dose Cadmium Aggravate the Injury of Mice Liver through Increasing Intestinal Permeability
Source: Microorganisms. 2020 Feb 5;8(2):211. doi: 10.3390/microorganisms8020211 (PMC7074735; doi:10.3390/microorganisms8020211)
Supplement: Supplementary file 1 [file microorganisms-08-00211-s001.pdf]

Table S1. Primers used for quantitative real-time PCR in this study

Gene	Sequence (5'-3")
ZO-1	F: ACTCCCACTTCCCCAAAAAC<br>R: CCACAGCTGAAGGACTCACA
JAM-A	F: CTGATCTTTGACCCCGTGAC<br>R: ACCAGACGCCAAAAATCAAG
Ocludina	F: ACTGGGTCAGGGAATATCCA<br>R: TCAGCAGCAGCCATGTACTC
Akk AM	F: CAGCACGTGAAGGTGGGGAC<br>R: CCTTGCGGTTGGCTTCAGAT
16S rRNA V3-V4	F: CCTACGGGAGGCAGCAG<br>R: ATTACCGCGGCTGCTGG
GAPDH	F: AGGTCGGTGTGAACGGATTTG<br>R: GGGGTCGTTGATGGCAACA
Acsf2	F: CAAATACTCCGAGTCCCT<br>R: CATCCTTCTGGGCTCTAT
Gm15922	F: GATGTCGTCGGTCTGTTC<br>R: CCTCGTAAATGCTCGTTA
Itgal	F: TGGAGGTTATTGCTGGTT<br>R: TCTGAATGGTGATGGGAG
Mvd	F: CAGAGGGCACAGACAAGC<br>R: ATGCAGGCGGTCATACAG
Fam35a	F: GCTCATACTTTGGCGGTGTT<br>R: GTGCTCCGCAGGGTTCAT

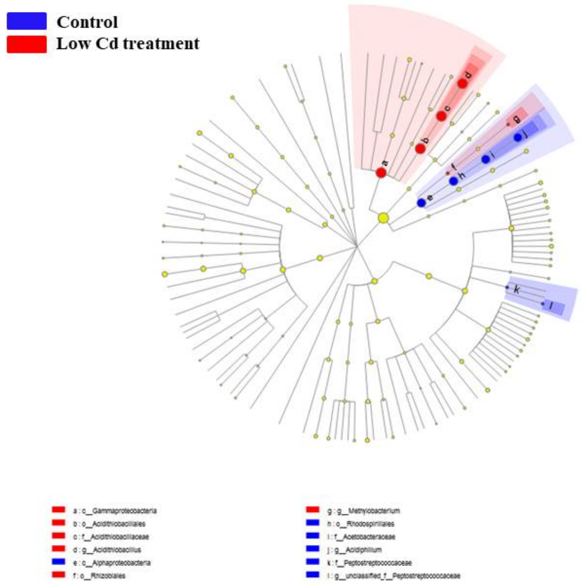

Figure S1. The enriched taxa in control and LDC fecal microbiota were represented in Cladogram. The central point represents the root of the tree (Bacteria), and each ring represents the next lower taxonomic level (phylum to genus: p, phylum; c, class; o, order;

f, family; g, genus). The diameter of each circle represents the relative abundance of the taxon.

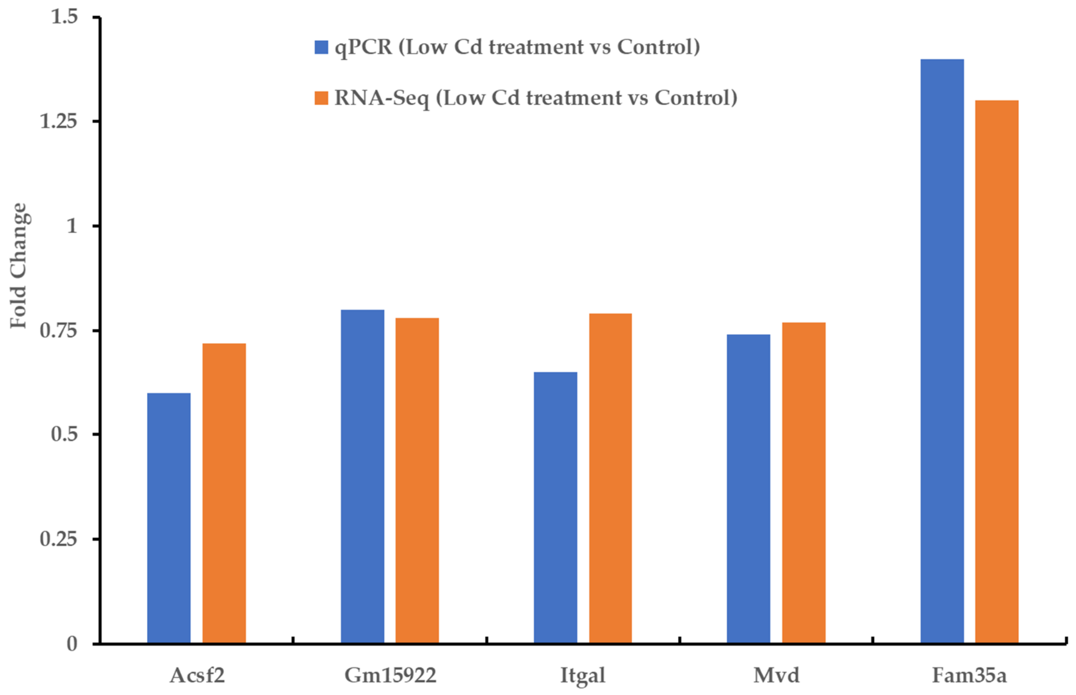

Figure S2. Quantitative real-time PCR verification of RNA-sequencing results. The x-axis represents genes, the y-axis represents the logarithm of fold change.

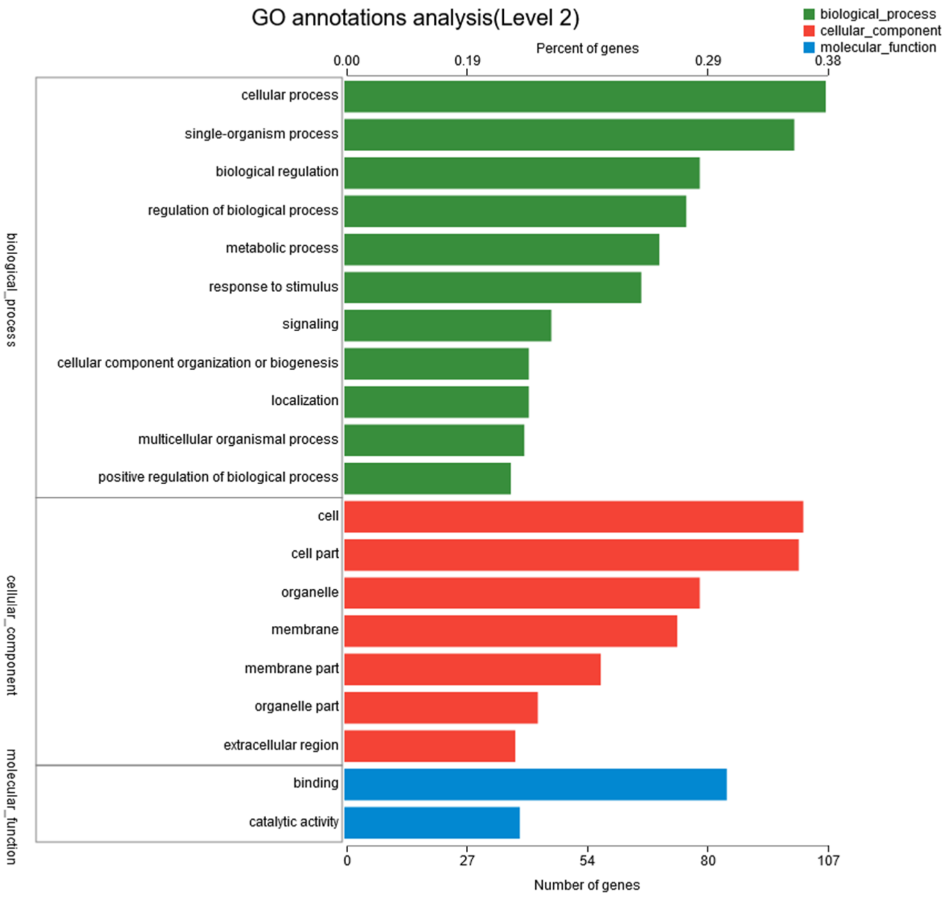

Figure S3. Histogram of Gene Ontology annotations of the differentially expressed genes between control and Low Cd treatment.
